# Supplementary material for: Alterations in Healthy Adult Canine Faecal Microbiome and Selected Metabolites as a Result of Feeding a Commercial Complete Synbiotic Diet with Enterococcus faecium NCIMB 10415
Source: Animals (Basel). 2022 Dec 30;13(1):144. doi: 10.3390/ani13010144 (PMC9817848; doi:10.3390/ani13010144)
Supplement: Supplementary file 1 [file animals-13-00144-s001.zip › Figure S1.pdf]

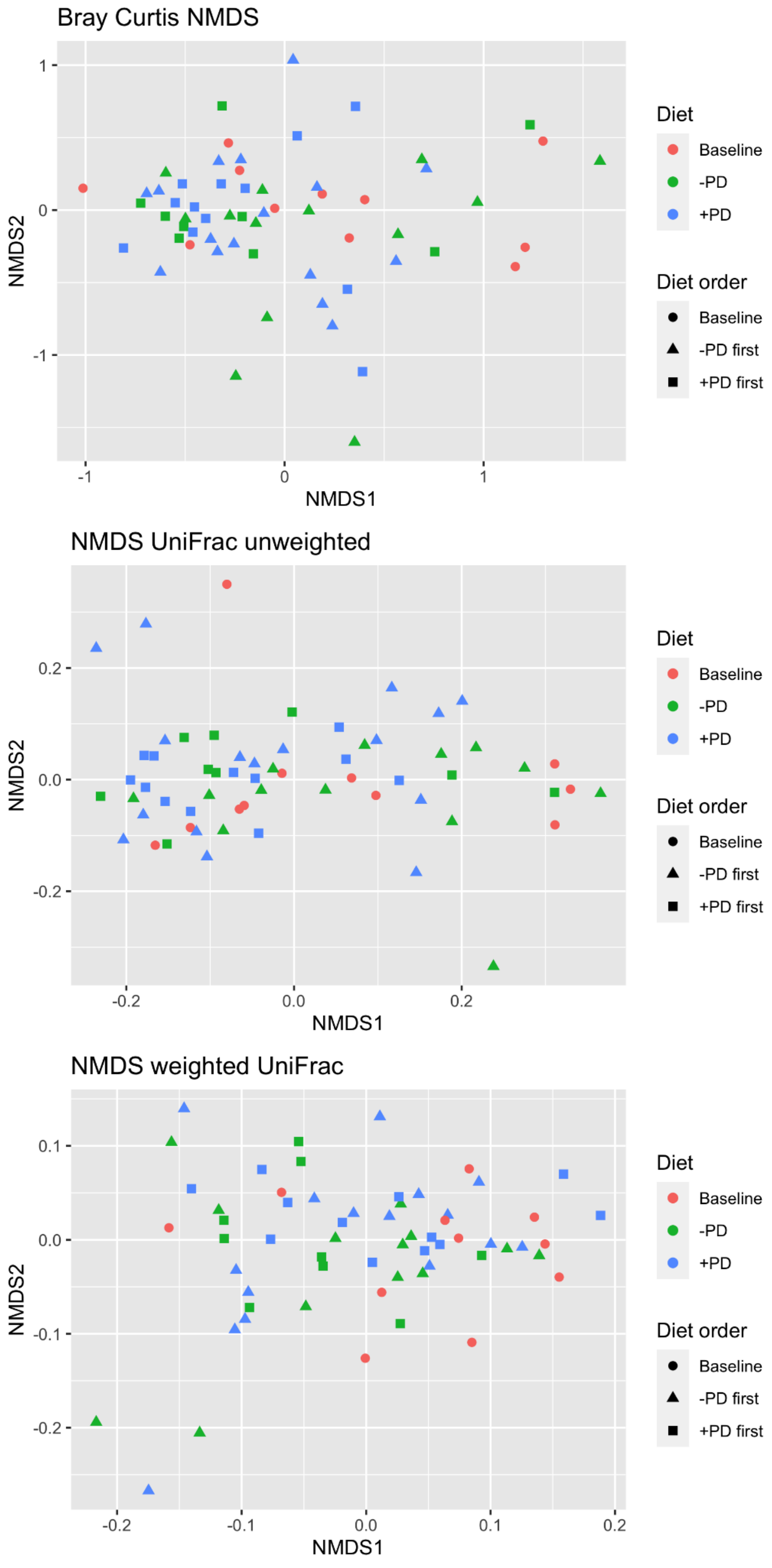

Figure S1. Microbiome beta diversity. Boxplot showing the beta diversity (specifically the Bray Curtis, unweighted UniFrac and weighted UniFrac) of the faecal microbiome when the study population was fed a diet manufactured with (blue) or without (green) *Enterococcus faecium* NCIMB 10415 as compared to baseline diversity at inclusion (red). Each colour on the plot identify the individual dog across diet and time.
