# Supplementary material for: Alterations in Healthy Adult Canine Faecal Microbiome and Selected Metabolites as a Result of Feeding a Commercial Complete Synbiotic Diet with Enterococcus faecium NCIMB 10415
Source: Animals (Basel). 2022 Dec 30;13(1):144. doi: 10.3390/ani13010144 (PMC9817848; doi:10.3390/ani13010144)
Supplement: Supplementary file 1 [file animals-13-00144-s001.zip › Table S1.pdf]

Table S1. All recruited dogs.

Laboratory references						6.50-18.10	3.2-12.1	0.0-1.2	1.00-4.80	0.00-1.20	0.00-0.05	4.6-8.4	200-500	6-102	19.8-174	3.9-6.55	3.3-9.4	40-130	3.50-6.99	0-5	26-44	2.20-3.30	0.63-1,05	0.91-1.96	142-154	3.8-5.1	106-123	
Dog	Breed	Sex	Age [years]	Body weight [kg]	Body condition score	WBC [10^9/L]	Neutrophils [10^9/L]	Monocytes [10^9/L]	Lymphocytes [10^9/L]	Eosinophils [10^9/L]	Basophils [10^9/L]	RBC [10^{12}/L]	Thrombocytes [10^9/L]	ALT [U/L]	ALP [U/L]	Glucose [mmol/L]	UREA [mmol/L]	Creatinine [umol/L]	Cholesterol [mmol/L]	Bilirubin [mmol/L]	Albumin g/L	Calcium [mmol/L]	Magnesium [mmol/L]	Phosphate [mmol/L]	Sodium [mmol/L]	Potassium [mmol/L]	Chloride [mmol/L]	Role in study
1	Giant Schnauzer	ME	3.0	46.3	5/9	9.86	5.79	0.39	3.07	0.57	0.02	6.73	188[d]	34	68	5.1	5.3	91	5.86	2	35	2.58	0.76	1.1	148	4.42	115	Completed study
2	Cocker Spaniel	ME	2.8	15.4	6/9	10.99	6.81	0.59	3.32	0.25	0.01	6.57	187[d]	29	54	4.7	6.0	80	7.23	2	36	2.82	0.73	1.2	148	4.49	112	Excluded due to diarrhoea.
3	Broholmer	ME	2.5	67	4/9	6.32	4.65	0.27	1.24	0.13	0.01	6.71	237	23	108	5.2	4.8	88	6.92	1	34	2.61	0.73	1.3	146	4.63	115	Excluded due to persistent weight loss
4	Broholmer	FE	4.0	43.5	5/9	7.69	4.69	0.60	1.96	0.41	0.01	6.32	366	47	223	4.8	5.1	76	10.04	2	37	2.64	0.99	1.4	147	4.88	114	Completed study
5	Mixed breed	MN	6.0	26.2	5/9	7.19	4.66	0.35	1.57	0.59	0.00	7.02	166[e]	192	41	5.4	5.1	80	6.49	1	35	2.65	0.82	1.4	148	4.35	114	Completed study
6	Field Trail Cocker Spaniel	MN	5.8	26.5	6/9	11.89	6.59	0.68	3.41	1.18	0.02	6.34	299	49	39	4.9	15.0	83	2.90	0	30	2.70	0.79	1.6	146	4.69	116	Completed study
7	Labrador retriever	ME	5.5	32.1	4/9	5.64	3.91	0.29	1.21	0.23	1.01	6.26	259	50	20	5.7	5.8	87	3.65	2	35	2.56	0.83	1.0	145	4.70	115	Completed study
8	Berger Blanc Suisse	ME	3.1	33.0	4/9	5.14	2.85	0.36	1.56	0.35	0.02	6.62	168[e]	119	33	5.0	8.3	126	6.65	1	33	2.78	0.85	1.4	150	4.19	116	Completed study
9	Border Collie	FE	3.1	18.1	6/9	6.09	4.26	0.32	1.04	0.47	0.00	6.38	318	49	62	6.0	5.8	104	6.39	1	33	2.36	0.85	1.1	150	4.15	119	Completed study
10	Mixed breed	ME	4.3	27.2	6/9	8.57	7.01	0.28	1.18	0.07	0.02	7.10	336	34	53	5.7	8.3	81	5.29	2	38	2.71	0.81	1.5	147	4.48	112	Excluded due to diarrhoea
11	West Highland White Terrier	ME	7.3	10.9	5/9	8.12	5.55	0.28	1.86	0.42	0.01	6.69	353	32	96	5.0	4.8	73	5.75	1	31	2.35	0.70	1.2	149	4.61	113	Completed study
12	Mixed breed	MN	3.9	45.8	4/9	6.22	3.51	0.39	1.68	0.61	0.02	7.1	222	28	68	4.2	6.6	83	5.37	2	37	2.71	0.80	1.1	145	4.28	114	Excluded at owners request due to increased appetite and decreased defecation frequency
13[a]	Czechoslovakian Wolfdog	FE	2.1	30	5/9	5.99	3.71	0.24	1.75	0.26	0.03	7.63	270	36	31	5.1	7.3	98	5.17	1	37	2.63	1.02	1.2	148	4.33	117	Excluded at owners request due to unconfirmed weight loss
14[a]	Czechoslovakian Wolfdog	FE	2.6	29.8	5/9	7.59	4.63	0.33	1.95	0.65	0.02	7.59	359	37	76	4.8	5.5	92	4.50	1	33	2.65	0.88	1.0	147	4.17	115	Excluded at owners request due to unconfirmed weight loss
15	Greyhound	MN	3.1	26	4/9	5.54	3.71	0.51	1.26	0.03	0.03	7.96	146[e]	51	70	5.6	4.6	135	2.83	1	33	2.62	0.89	1.3	150	4.08	117	Completed study
16	Border Collie	FE	4.5	17.6	5/9	4.85	3.15	0.26	1.17	0.23	0.02	5.90	296	33	81	4.3	5.8	96	5.57	2	27	2.33	0.7	1.0	148	4.56	115	Completed study
17	Mixed breed	MN[f]	3.1	36.8	5/9	6.21	2.93	0.30	2.20	0.75	0.03	7.23	263	38	310	4.9	5.5	102	8.13	1	30	2.47	0.68	1.4	145	5.02	111	Completed study

e1[b]	Collie	FE	6.0	30.8	6/9	9.42	7.75	0.41	1.05	0.21	0	5.41	377	69	47	6.0	4.5	68	7.38	0	30	2.65	1.37	1.3	147	4.20	113	Excluded due to hypermagnesemia
e2[b]	Cocker Spaniel	ME	6.1	16.9	5/9	8.19	6.57	0.27	1.03	0.30	0.01	7.00	197[d]	32	105	4.1	6.0	74	5.89	2	35	2.65	1.39	1.2	148	4.21	112	Excluded due to hypermagnesemia
e3[b]	Collie	FE	3.1	18.6	5/9	7.59	5.86	0.43	1.07	0.22	0.01	5.98	196[e]	53	22	4.5	13.2	174	7.40	0	36	2.97	1.41	1.1	152	4.26	111	Excluded due to hypermagnesemia and azotemia
e4[b]	Mixed breed	ME	2.1	29.2	6/9	7.20	3.82	0.22	1.66	1.51	0.00	6.72	179[e]	45	106	5.1	5.7	105	5.51	1	37	2.72	1.30	0.9	150	4.43	117	Excluded due to hypermagnesemia, hypophosphatemia and eosinophilia
e5[b]	Australian Cattle Dog	FE	6.1	22.7	5/9	4.32	2.73	0.20	1.16	0.22	0.01	6.35	114[d]	31	123	4.8	2.8	81	5.73	1	36	2.84	1.35	1.0	150	4.32	115	Excluded due to hypermagnesemia
e6[c]	Samoyed	FE	2.7	18.6	4/9	14.68	8.49	0.97	2.92	2.24	0.05	7.51	354	31	59	5.6	5.3	66	5.00	1	32	2.67	0.90	1.2	149	4.40	117	Exclude due to eosinophilia
e7[c]	Samoyed	FN	2.7	20.0	4/9	11.7	6.51	0.47	2.21	2.49	0.01	5.29	276	42	72	4.3	6.5	83	3.81	2	32	2.58	0.92	1.4	150	4.33	116	Exclude due to eosinophilia
e8[c]	Samoyed	ME	2.7	25.5	4/9	15.62	10.5	0.68	2.40	1.99	0.03	6.06	283	34	70	4.4	7.9	87	5.09	1	32	2.42	0.93	1.6	152	4.47	116	Exclude due to eosinophilia
e9[c]	Swedish Lapphund	ME	2.7	12.9	4/9	9.56	5.96	0.42	1.96	1.17	0.03	6.20	260	40	805	5.6	5.5	95	3.71	3	34	2.45	0.79	1.5	149	4.57	117	Excluded due to elevated alkaline phosphatase

Green labeled dogs completed the study. Yellow labeled dogs were initially included in the study but excluded again during the study course. Red labeled dogs were recruited, but not included in the study. ME: male entire, MN: male neutered, FE: female entire. FN: female neutered.

[a] Same owner. Weight loss compared to time of inclusion was noted at a different set of scales by the owner, who hereafter requested withdrawal from the study.

[b] Same owner. No dogs were enrolled in the study due to hypermagnesemia.

[c] Same owner. No dogs were enrolled in the study due to eosinophilia (n=3) and elevated alkaline phosphatase (n=1).

[d] Thrombocyte aggregates, thrombocyte number interpreted as within reference levels.

[e] A few macrothrombocytes evaluated on blood smear.

[f] Chemically neutered 4 months prior to inclusion.
