# Supplementary material for: Alterations in Healthy Adult Canine Faecal Microbiome and Selected Metabolites as a Result of Feeding a Commercial Complete Synbiotic Diet with Enterococcus faecium NCIMB 10415
Source: Animals (Basel). 2022 Dec 30;13(1):144. doi: 10.3390/ani13010144 (PMC9817848; doi:10.3390/ani13010144)
Supplement: Supplementary file 1 [file animals-13-00144-s001.zip › Table S2.pdf]

Table S2. Diets and supplements fed to the dogs prior to study enrolment

Dog	Breed	Previous diet	Supplements	Treats	Other
1	Giant Schnauzer	Royal Canine Giant breed	Omega 3	Yes	NA
4	Broholmer	Spanish kibble Diet	No	NA	Will sometimes eat horse manure and apples
5	Mixed breed	Pedigree	No	Yes	Will sometimes eat horse and cattle manure and carrots
6	Field Trail Cocker Spaniel	Olivers Pet Food kibbled diet	No	Yes	Chewing bone. Occasionally grass and sand
7	Labrador retriever	Royal Canine Adult	No	Yes	Occasionally grass. Leftovers
8	Berger Blanc Suisse	Eukanuba adult Chicken and Rice	No	Yes	No
9	Border Collie	Royal Canine medium adult	Salmon oil, vitamins	Yes	No
11	West Highland White Terrier	Eukanuba Westie	No	Yes	Leftovers
15	Greyhound	Changing brands of kibbled diet	Salmon oil	Yes	NA
16	Border Collie	Arion varying between type	No	Yes	NA
17	Mixed breed	Eukanuba	No	Yes	Leftovers
